# CD8^+^脾脏γδT细胞淋巴瘤1例

**DOI:** 10.3760/cma.j.issn.0253-2727.2023.02.018

**Published:** 2023-02

**Authors:** 桐 陆, 丹 伊, 春卉 金, 银萍 王, 丽梅 曲

**Affiliations:** 吉林大学第一医院病理科，长春 130021 The Pathological Department, the First Hospital of Jilin University, Changchun 130021, China

患者，女，46岁，因“皮肤散在瘀点1个月，加重伴乏力、饱腹感20余天”于2015年6月就诊于吉林大学第一医院。入院前一周患者就诊于当地医院考虑为急性淋巴细胞白血病。入院后骨髓穿刺活检显示：骨髓增生活跃。粒细胞系：比例减低（占18％），各阶段粒细胞比例减低，形态未见明显异常。红细胞系：比例减低（占9.5％），有核红细胞及成熟红细胞形态未见明显异常。淋巴细胞比例增多（占72.5％），其中原始淋巴细胞占67％，形态上胞体大小不等，胞质不规则，核染色质较粗，可见核仁1～2个。全片未见巨核细胞，血小板少见。POX染色：病理细胞100％阴性。骨髓流式细胞术检查：成熟T淋巴细胞（倾向于TCRγ/δ型）占有核细胞34.96％，考虑T细胞淋巴瘤可能性大。病毒血清学显示EB病毒核心抗原IgG抗体1.614，衣壳抗原IgG抗体5.256。肝功能：AST 75.1 U/L，ALT 50.8 U/L，γ-谷氨酰转肽酶61.0 U/L，ALP 117.2 U/L，LDH 1 343 U/L。查体：全身可见散在瘀点，以双侧腋下、后背为著。浅表淋巴结未触及肿大。肝脾肋缘下可触及，肝肋缘下2 cm，脾肋缘下1.5 cm。患者全腹CT检查提示脾脏多发占位。PET-CT检查示：肝、脾体积明显增大伴代谢弥漫增高；腰5椎体密度增高，骨骼代谢弥漫稍高。排除手术禁忌后行腹腔镜脾切除术。病理检查：送检脾脏组织，总体积约18 cm×15 cm×6 cm。显微镜下可见脾红髓内充满形态单一、中等大小的淋巴细胞，核圆形或卵圆形，染色质稀疏，核仁不明显，核分裂象不易识别。免疫组化结果显示：Ki-67（50％+），CD2（+），CD3（+），CD5（−），CD7（+），CD4（−），CD8（+），CD43（+），CD56（+），TIA-1（+），CD20（−），TdT（−），PAX-5（−），CD34（−），CD10（−），CD1a（−），MPO（−），CD99（−），Granzyme B（−），EMA（−），Bcl-6（−），CD21（−），PD-1（−），ALK（−），CD30（−），原位杂交检测EBER（−）。分子病理显示TCRγ的Vγ1-Vγ10-Jγ（+）、Vγ9-Vγ11-Jγ（+），TCRδ的Vδ-Dδ2-Jδ-Dδ3（+），见基因重排单克隆。病理诊断：脾γδT细胞淋巴瘤。患者确诊后即采用CHOP方案，化疗3个疗程后开始采用Hyper CVAD A方案，化疗间期骨髓、外周血流式细胞术提示复发后，更改为Hyper CVAD B方案。随着病情进展患者出现胸部不适，考虑与贫血有关。全身淋巴结未见肿大，病变累及肝脏、脾脏及骨髓。患者因一般情况差放弃化疗，于确诊后8个月死亡。

讨论：肝脾T细胞淋巴瘤（HSTCL）是一种罕见的高度侵袭性外周T细胞淋巴瘤亚型。其临床表现主要为肝脾大和血小板减少，淋巴结肿大少见，可早期累及骨髓。其发病机制与病因尚不明确，治疗也缺乏一线方案，现有数据多提示联合化疗的必要性。按其TCR受体基因重排分为γδ和αβ两个亚型，其中γδT细胞淋巴瘤类型更为多见。国外有研究者通过分析28例HSTCL患者发现αβ TCR表达升高者预后更差。大约20％的HSTCL发生于存在免疫抑制或免疫失调的患者。也有报道表明HSTCL与EB病毒感染相关，而且可能加速HSTCL的临床进展，导致预后差。本例患者病毒血清学测定表明存在EB病毒感染，虽然肿瘤细胞EBER检测阴性，但推测患者发病与此相关并导致较差预后。HSTCL常起源于外周γδ型T细胞。在健康成人中，γδT细胞不足循环T细胞总数的5％，在人体组织中通常分布于淋巴样组织、黏膜组织、皮肤组织和脾脏的红髓，来自骨髓中的双阴性CD4^−^/CD8^−^胸腺前体，故肝脾γδT细胞淋巴瘤患者的免疫表型大部分为：CD2、CD3、CD7、TCRγδ（+），不表达CD4、CD5和CD8；少数病例表现为CD4^−^/CD8^+^，相关预后暂不明确。研究显示，大多肿瘤性γδT细胞是非活化的细胞毒细胞，即TIA-1阳性，而颗粒酶B和穿孔素为阴性。本例患者免疫组织化学表达为CD4^−^/CD8^+^，属于罕见表型，综合考虑其典型临床表现和肿瘤细胞的窦内浸润特征，符合肝脾γδT细胞淋巴瘤的诊断。

